# Ununited Fractured Rib — Successfully Treated by an Operation

**Published:** 1856-03

**Authors:** 


*Ununited Fractured Rib—successfully treated by an Operation.* By Prof. PAUL F. EVE, M. D., of Nashville, Tennessee.

On the 11th of last November, 1854, I operated before the class of our University, upon Mr. Wm. Briant, of Bledsoe county, East Tennessee, who some two years before had received an injury by a fall, having at the time a heavy burden on his shoulder. The result was a fracture of one or more ribs; and he came to our college clinic on account of a continual discharge of pus from sinuses situated over the eighth rib of the left side, a little anterior to its middle.

The patient, with this exception, enjoyed good health, was a stout, active, laborious man, and possessed an excellent constitution. A probe introduced into the sinuses having detected denuded bone, it was proposed to convert the issues into one by an incision which would also expose the true condition of the diseased parts. No looser portion was found, but the fractured extremities of the ribs were enveloped in an abscess, and removed with stout forceps; the wound was dressed, leaving a tent, made of patent-lint, at the bottom, with adhesive plasters.

The broken ends of the ribs were twisted off with strong forceps, instead of employing cutting instruments for this purpose.

A few days after the operation, the patient returned home with injunctions to wear the tent deep in the wound, so that it might act as a seton, for several months between the broken ends of the bone.

The 10th of June, 1855, seven months after he left Nashville, Mr. B. wrote me, that "the side you operated upon last November is sound and well, and I enjoy fine health, and can work and attend to my business as heretofore." Two months later, in August last, my colleague, Prof. Watson, examined the case, found it as represented by the patient himself, and pronounced it perfectly healed.

It is probable a seton between the fractured ends of the rib might have produced the same happy result.—*Nashville Jour. of Med. and Surg.*

